# Tensile Behavior of High-Strength, Strain-Hardening Cement-Based Composites (HS-SHCC) Reinforced with Continuous Textile Made of Ultra-High-Molecular-Weight Polyethylene

**DOI:** 10.3390/ma13245628

**Published:** 2020-12-10

**Authors:** Ting Gong, Iurie Curosu, Frank Liebold, Duy M. P. Vo, Konrad Zierold, Hans-Gerd Maas, Chokri Cherif, Viktor Mechtcherine

**Affiliations:** 1 Institute of Construction Materials, Technische Universität Dresden, 01062 Dresden, Germany; ting.gong@tu-dresden.de (T.G.); viktor.mechtcherine@tu-dresden.de (V.M.); 2 Institute of Photogrammetry and Remote Sensing, Technische Universität Dresden, 01062 Dresden, Germany; frank.liebold@tu-dresden.de (F.L.); hans-gerd.maas@tu-dresden.de (H.-G.M.); 3 Institute of Textile Machinery and High Performance Technology, Technische Universität Dresden, 01062 Dresden, Germany; duy.vo@tu-dresden.de (D.M.P.V.); konrad.zierold@tu-dresden.de (K.Z.); chokri.cherif@tu-dresden.de (C.C.)

**Keywords:** SHCC, ECC, textile reinforcement, short-fiber reinforcement, hybrid reinforcement, UHMWPE, tension, pullout

## Abstract

The paper at hand presents an investigation of the tensile behavior of high-strength, strain-hardening cement-based composites (HS-SHCC), reinforced with a single layer of continuous, two-dimensional textile made of ultra-high molecular weight polyethylene (UHMWPE). Uniaxial tension tests were performed on the bare UHMWPE textiles, on plain HS-SHCC, and on the hybrid fiber-reinforced composites. The bond properties between the textile yarns and the surrounding composite were investigated in single-yarn pullout experiments. In order to assess the influence of bond strength between the yarn and HS-SHCC on the tensile behavior of the composites with hybrid fiber reinforcement, the textile samples were analyzed both with, and without, an additional coating of epoxy resin and sand. Compared to the composites reinforced with carbon yarns in previous studies by the authors, the high elongation capacity of the UHMWPE textile established the higher strain capacity of the hybrid fiber-reinforced composites, and showed superior energy absorption capacity up to failure. The UHMWPE textile limited the average crack width in comparison with that of plain HS-SHCC, but led to slightly larger crack widths when compared to equivalent composites reinforced with carbon textile, the reason for which was traced back to the lower Young’s modulus and the higher elongation capacity of the polymer textile.

## 1. Introduction

Concrete and reinforced concrete (RC) structures feature relatively low resistance to various types of dynamic loading, such as earthquakes, impacts, or blasts, which makes it important to develop effective strengthening solutions for existing critical infrastructure. Within the framework of the multidisciplinary Research Training Group GRK 2250/1 “Mineral-bonded composites for enhanced structural impact safety”, high-performance composites are developed and investigated as externally applied, impact-resistant strengthening layers [1]. The material basis for the developed strengthening layers is provided by textile reinforced concrete (TRC) [2,3,4,5] and strain-hardening cement-based composites (SHCC) [6,7,8]. Aside from their suitable mechanical properties and high durability, the high mechanical and physical compatibility with the concrete substrate and the small but effective layer thickness of up to 20 mm, in combination with feasible application techniques by spraying or lamination, make these composites highly promising for enhancing structural impact resilience.

TRC, also termed textile reinforced mortar (TRM), consists of fine-grained, cementitious matrices reinforced by continuous two- or three-dimensional textile meshes, usually made of carbon or glass multifilament yarns, and exhibits ductile, strain-hardening tensile behavior [9,10,11,12]. With regard to cyclic and highly dynamic loading, the geometric configuration of typical 2D and 3D textile reinforcements is not sufficiently fine to ensure dense crack patterns and high energy dissipation capacity, without substantial fragmentation and spalling of the cementitious matrix [13]. The latter can be mitigated by the addition of short, dispersed fibers. Given the requirements for high energy dissipation through large inelastic deformations, in conjunction with small layer thicknesses, the application of high-performance polymer micro-fibers, according to the micromechanical design principles of SHCC, and also engineered cementitious composites (ECC), provides a promising set of advantages.

In combination with an adequate mineral-bonded matrix, polymer micro-reinforcement can increase the first-crack stress of the composites considerably, and ensure desirable micro-confinement, crack-bridging, and pronounced multiple cracking under advancing deformations [14]. In this context the combination of SHCC with textile reinforcement, and in conjunction with targeted material design, represents a promising and viable solution. Although the PE fibers exhibit a relatively low melting temperature of approximately 150 °C [15,16,17], which may limit the applicability of such textiles, the positive rate sensitivity of these fibers with regard to their tensile strength and Young’s modulus [18,19] represents a promising feature for applications involving dynamic loading. The targeted material design should account for the complex micro- and meso-mechanical interactions in these composites, for the pronounced rate sensitivity of SHCC [20,21], and for the textile reinforcement itself [22,23,24].

The synergetic action of various HS-SHCCs, with different types of carbon textile, has been investigated in previous studies by the authors under quasi-static and impact tensile loading [25,26]. It has also been shown that the mechanical properties and coating of the textile reinforcement define to a large extent the tensile strength and the pre-peak deformability of the hybrid fiber-reinforced composites under investigation. The carbon textile exhibits high tensile strength and stiffness, both of which are essential for the strong confinement of the RC elements, both under quasi-static and severe dynamic loading [1,13]. However, the strain capacity of typical HS-SHCC is potentially higher compared to that of carbon yarns [26]. Thus, it is of interest to assess the potential of high-strength mineral-bonded composites with hybrid fiber reinforcement in the range of extremely high deformations. The aim of such an extensive parameter study is to form a solid basis for subsequent optimizations, targeting various performance requirements, and involving sustainability factors.

Textiles made of polymers such as aramid, poly, p-phenylene-2,6-benzobisoxazole (PBO), and polypropylene (PP) exhibit relatively large elongation capacities [22]. However, PP fibers exhibit relatively low tensile strength and Young’s modulus, while the durability of the aramid and PBO fibers in cementitious environments has still not been fully explored. In the investigation presented here, ultra-high molecular weight polyethylene (UHMWPE, short: PE) was studied as a textile reinforcement. This was motivated by the appropriate mechanical properties of such textiles. The elongation capacity of PE fibers is approximately 3.5%, which is more than twice that of carbon fiber. Furthermore, the elongation capacity of PE is comparable to the strain capacities of high-strength SHCC, as assessed by the authors [26] and reported elsewhere in the literature [27].

The series of experimental investigations presented by the authors is a continuation of previous studies in which carbon textiles were investigated [25,26,28,29]. Carbon textiles with coatings (also impregnations) consisting of acrylate [26,30], styrene-butadiene [25,26,28,29], and epoxy [30] have been upscaled to the industrial level, and are commercially available. In this study, PE textiles were produced and impregnated, according to the same technology used on the commercially available carbon textile, with styrene-butadiene coating [26]. What is more, in order to assess the influence of the bond strength between textile and SHCC on the tensile behavior of the hybrid fiber-reinforced composites, the PE textile was investigated both with, and without, an extra coating, which consisted of epoxy resin and sand. Unlike industrially applied epoxy coating, serving also as impregnation, and filling the inter-filament space [30], the epoxy and sand coating in this study was applied manually with a brush, and it only covered the outer surface of the PE yarns.

The experimental study as presented consisted of single-yarn pullout tests as well as tension experiments on PE textile specimens, on plain HS-SHCC, and on hybrid fiber-reinforced composites. The multiple cracking of the composite specimens under tension was assessed by means of optical measurements, and statistically evaluated with a digital image correlation (DIC) code, developed in-house [31,32,33].

## 2. Materials under Investigation

### 2.1. Two-Dimensional PE, Non-Crimp Textile

As opposed to the carbon textiles presented in the previous works by the authors [25,26,29], the PE textile investigated in this work is not commercially available. Indeed, it was developed by the Research Training Group GRK 2250/1 specifically for these investigations [1]. The PE textile was produced at the Institute of Textile Machinery and High-Performance Material Technology (ITM), at the Technische Universität Dresden, Germany. The PE fiber was provided by DSM, the Netherlands, under the brand name Dyneema SK75. The tensile strength of this fiber grade is in the range of 3.3 to 3.9 GPa [15]. Note, however, that the tensile strength of multifilament yarns is lower than that of the single filaments. The production followed the same technology, and targeted similar geometric properties as those of the standard and commercially available carbon textiles investigated in the previous works [26]. The average yarn counts and the effective yarn cross-sectional areas of the reference carbon textile TUDALIT-BZT2-V.FRAAS in the warp and weft directions are 3300 and 800 tex, 1.8 and 0.451 mm^2^, respectively [34]. Since the PE yarns were delivered in spools with a yarn count of 264 tex, the warp PE yarns in the produced textile were assembled from eight combined as-received PE multifilament yarns, while the weft yarns consisted of two as-received yarns. This restriction related to the yarn count on the provided spools resulted in a certain cross-sectional difference of the PE yarns compared to the previously studied carbon textile; see Table 1.

Production was performed on a biaxial stitch-bonding machine, Malimo 14,022 P2-S2, with a parallel weft insertion system. Such a machine was specially designed by KARL MAYER Technische Textilien GmbH (Obertshausen, Germany), and later significantly modified at ITM for the manufacture of non-crimp reinforcing fabrics with minimal yarn damage. In non-crimp fabrics anisotropic, high-performance multi-filament yarns are positioned straight, in various directions on the fabric surface, allowing for optimal exploitation of their mechanical properties and reinforcing performance. The different yarn systems are stitch-bonded using fine knitting threads, which are textured polyester (PES) filament yarns (7.6 tex) as commonly used in carbon fiber (CF) textile reinforcement.

**Figure 1:**
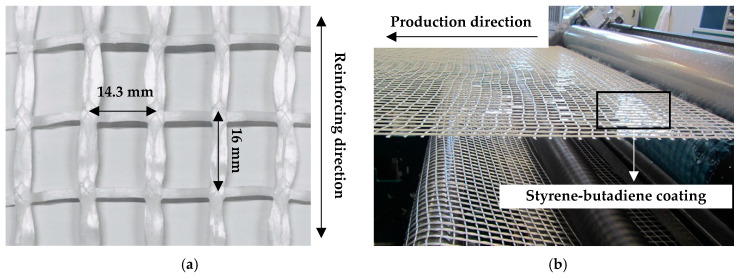
Production of PE textile: (**a**) geometry of the textile mesh, and (**b**) application of the styrene-butadiene coating.

In stitch-bonding, machine configuration and technological features have a significant influence on the product parameters attainable. Specifically, warp density results from machine gauge and yarn arrangement. The available stitch-bonding machine had a F7 gauge, referring to the seven sets of working elements, comprised of knitting threads, needles etc., per inch. Consequently, the realistic warp distance in the resulting textile must be a multiple of 3.6 mm. A warp distance of about 14.3 mm was chosen to approximate the 12.7 mm warp distance of the reference carbon textile. Accordingly, warp yarns and knitting threads were fed through only every fourth guider. On the other hand, weft density is a function of the forward speed of the transport chains and the fabric take-up speed. Since these technological parameters can be flexibly adjusted, a weft distance of 16 mm, equal to that of the reference carbon textile, was achieved on stitch-bonded PE textile reinforcement; see Figure 1a.

The textile coating is necessary to provide proper composite action among the individual filaments and an adhesive bond of the yarns to the cementitious matrices. The same coating material as that of the reference carbon textile was used, which is a watery styrene-butadiene based dispersion, named SBR Lefasol VL90/2, by Lefatex-Chemie GmbH, Brüggen, Germany. The coating of the stitch-bonded PE textile was performed offline on a multifunctional system, from Coatema Coating Machinery GmbH (Dormagen, Germany), using the Foulard method; see Figure 1b. The textile was drawn over a 40 m-long distance at a speed of 1 m/min and a curing temperature of 70 °C, which was intentionally restricted below the melting range of the PE yarn material treated. This setup allowed for an effective, process-reliable drying effect, while preventing the alteration of the textile’s mechanical properties after coating.

**Figure 2:**
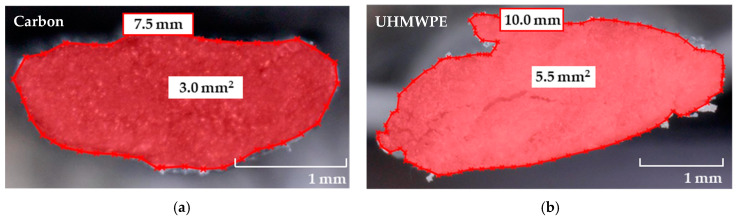
Characteristic cross-sectional features of (**a**) carbon and (**b**) PE multifilament yarns.

The cross-sectional shape of the warp yarns is presented, as a comparison with that of a reference carbon textile, in Figure 2. The yarns were cut into short segments and glued to a supporting frame, in this way ensuring their verticality on the microscope stage. The circumference and the cross-sectional area were derived using a digital microscope, Keyence VHX-6000 (Osaka, Japan). As presented in Figure 2b, the outer perimeter of the weft yarn of the PE textile was approximately 10 mm, while the bulk cross-sectional area was 5.5 mm^2^. Compared to the commercially available carbon textile, the relative arrangement of the PE filaments in the yarn was less compact.

**Figure 3:**
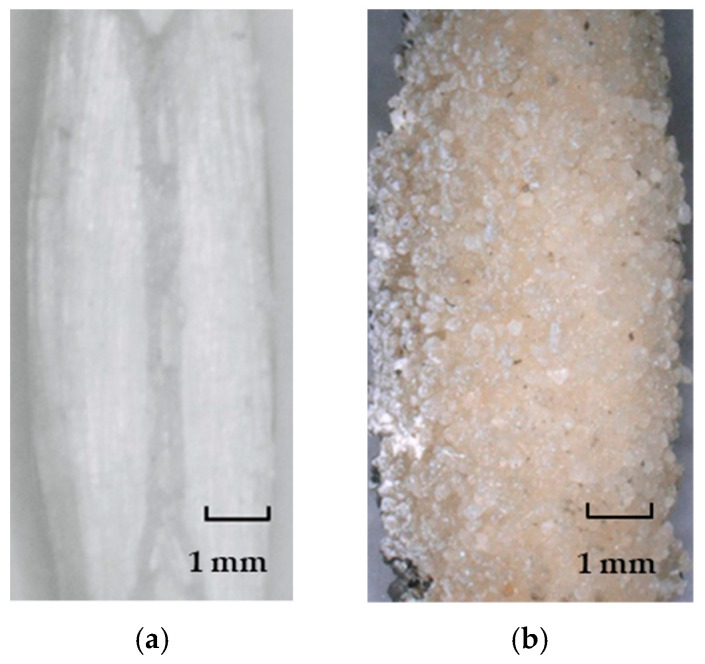
Surface appearance of the warp PE yarns under investigation: (**a**) in reference state, and (**b**) with epoxy resin and sand coating.

Based on previous investigations by the authors on carbon textiles [25,26], the styrene-butadiene impregnation ensures relatively low adhesion and, in turn, frictional bond to the cementitious matrix. In order to assess the influence of the bond strength between textile yarns and SHCC on the tensile behavior of the hybrid fiber-reinforced composites, an additional coating of epoxy resin and sand was applied manually to a separate batch of PE textiles. Epoxy resin 300 with hardener 3018 from BEHNKE Epoxidharze (Schöneiche, Germany) was used. The sand applied was from Quarzwerke GmbH (Hohenbocka, Germany), and had a particle size distribution between 0.125 and 0.5 mm. The additional coating entailed first the application of epoxy using a pencil, and the subsequent immersion of the textiles in a sand container. The surface properties of the clean and extra-coated PE yarns are presented in Figure 3a,b, respectively.

### 2.2. High-Strength SHCC

To enable a direct comparison with the previous studies involving carbon textile [26], the established high-strength SHCC composition was investigated in this work. The rather typical mixture design for such composites was developed and described in detail by the authors in previous publications [14,20,21]. The average compressive strength of this mixture at the age of 14 days is approximately 140 MPa [20,21]. As presented in Table 2, this SHCC contains a high dosage of cement and silica fume, and a relatively small content of fine quartz sand as fine aggregates. Given the high content of fines and the low water-to-binder-ratio of 0.18, a relatively high content of superplasticizer had to be used to ensure proper workability and homogeneity, in terms of fiber distribution and flaw content. The short reinforcing fibers were made of PE, produced by DSM, the Netherlands. The strength grade of the 6 mm-long PE fibers was SK62 [15], and their volume content in SHCC was 2%.

The 6 mm length of the fibers ensured proper crack-bridging, due to the strong anchorage in the high-strength matrix, and is smaller than the openings of the textile meshes. This is important in facilitating fiber penetration through the textile and transverse (out-of-plane) crack control, in this way avoiding scabbing under impact or matrix delamination along the textile mesh.

In this work, the PE textiles with, and without, epoxy resin and sand coating are designated as PE-E and PE, respectively. The respective combinations between the two textile variations and the high-strength SHCC are called SHCC-PE-E and SHCC-PE.

## 3. Experimental Program

### 3.1. Specimen Production and Setup for Single-Yarn Pullout Experiments

**Figure 4:**
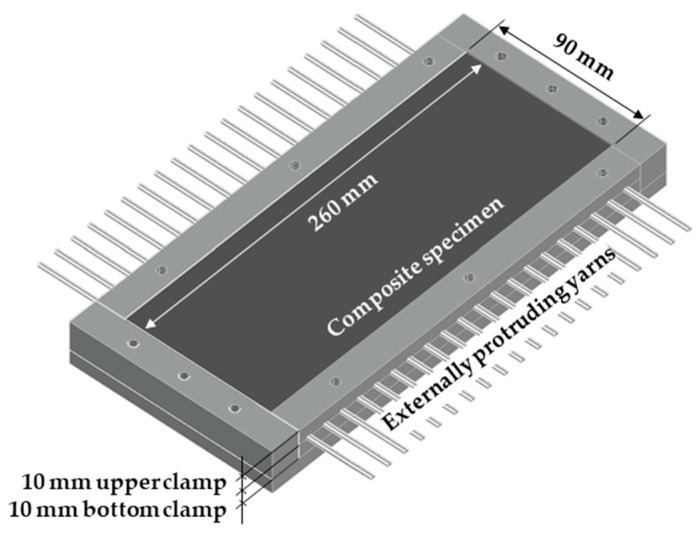
Schematics of the mold and specimen preparation for single-yarn pullout experiments. The textile is centered horizontally in the mold’s depth.

The production of the single-yarn pullout specimens started by casting plates with dimensions of 260 mm × 90 mm × 20 mm in a specially designed mold, as described in [26,29]. The first layer of SHCC was cast in the molds uniformly. The textile layer was subsequently placed on top and gently pressed, in order to squeeze the bottom SHCC upwards through the textile mesh. Finally, the top layer of SHCC was cast followed by leveling and smoothening. The PE yarns protruded outside of the molds and were clamped by the top plastic pieces in the middle of the plate thickness; see Figure 4. The plates were demolded 24 h after casting, sealed in plastic sheets, and stored for 27 days in a climatic chamber, with constant temperature of 20 °C and relative humidity of 65%.

**Figure 5:**
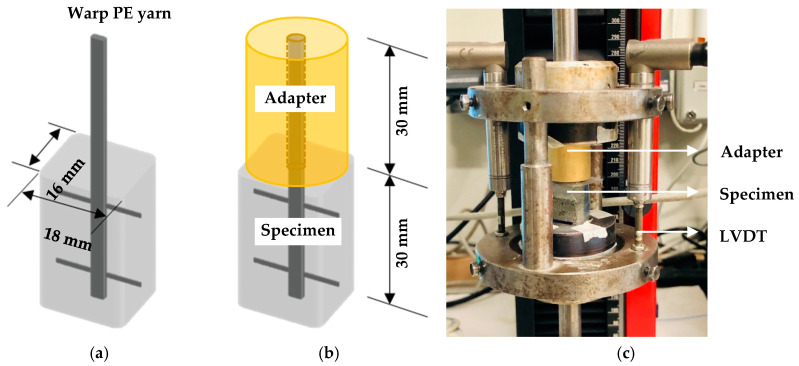
(**a**) Specimen geometry, (**b**) specimen with adapter, and (**c**) testing assembly for single-yarn pullout.

Prior to the mechanical testing small specimens were cut from the plate with dimensions and shape as shown in Figure 5a. The length of the HS-SHCC piece was 30 mm, and it defined the embedded length of the yarns. The length of the protruding yarns was 30 mm, and these were glued into brass adapters as shown in Figure 5b. Subsequently, the specimen assembly was glued at both ends in special steel rings bolted to aluminum stamps. The latter were rigidly fixed in the testing machine through threaded steel rods, as shown in Figure 5c. This testing configuration was originally developed for uniaxial tension experiments on cylindrical SHCC specimens [21], whereas the specimen assembly is also suitable for dynamic pullout experiments in a split Hopkinson tension bar [35]. Besides fixing the specimens, the adapters served as support for a steel frame, on which two linear variable differential transducers (LVDTs) were installed directly on the specimen to measure the displacement of the yarns, in this way making the measurements independent of the compliance of the testing machine. Note, however, that the LDVT measurements do not exclude possible deformations of the assembly itself, e.g., adapter and steel rings. The pullout experiments were performed in an electromechanically actuated testing machine, Zwick Roell 1445 (Ulm, Germany), at a displacement rate of 0.05 mm/s.

### 3.2. Production of In-Situ Textile Specimens for Tensile Testing

**Figure 6:**
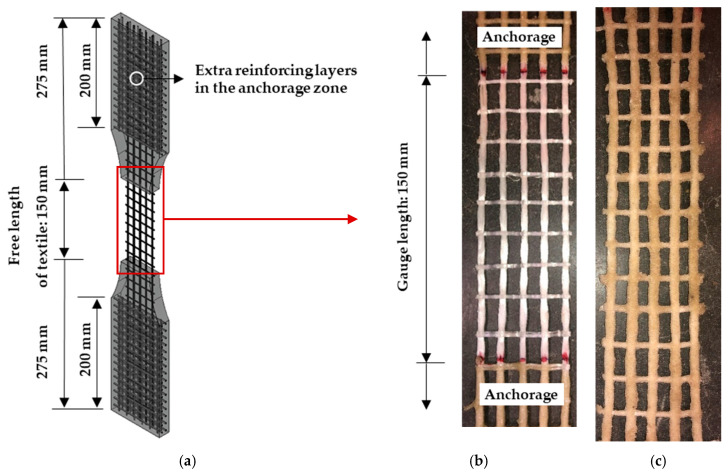
(**a**) Textile specimen configuration with the anchorage pieces, (**b**) textile without additional coating in the gauge portion, and (**c**) textile with epoxy resin and sand coating also in the gauge portion.

The tensile properties of the textiles may differ substantially from the tensile behavior of the single PE filaments, and indeed should be assessed in conditions identical to those in the cementitious composites. Designed and produced for this purpose, special textile specimens implied anchorage inside the HS-SHCC, as shown in Figure 6a. In addition, these specimens are geometrically equivalent to the composite specimens presented in the next section, and allow for a comparative analysis of the experiments on bare textiles and on cementitious composites both with and without textile reinforcement.

For the paper at hand, the textile stripes were stretched and fixed at both ends with clamps during the application of the epoxy resin and sand. This was performed to achieve a regular mesh shape within the 150 mm-gauge length; see Figure 6b,c. What is more, the anchorage portions of the textile were also coated to avoid slippage from the anchorage parts of the loaded specimens. To avoid damage and cracking in the anchorage pieces due to the mechanical clamping, additional textile stripes made of carbon were placed in the anchorage regions, as shown in Figure 6a and described in previous works [26,29]. The total length of the specimens was 700 mm, with a 20 mm thickness of the anchorage parts. The gauge length was 150 mm, and consisted of five warp yarns in the reinforcing direction.

**Figure 7:**
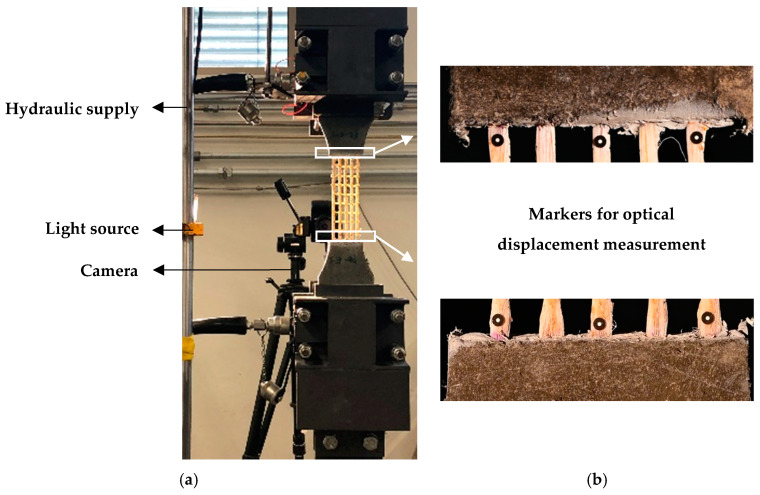
(**a**) Testing setup with a textile specimen, and (**b**) markers for optical displacement measurements.

The uniaxial tension tests of the textile specimens were performed using an Instron 8501 (Norwood, MA, USA) hydraulic testing machine at a displacement rate of 0.05 mm/s. The specimens were clamped within specially designed steel plates, with a pressure of approximately 10 MPa generated by hydraulic jacks. The clamping surface at each end was 150 cm^2^, with knurled inner surfaces to ensure the specimens’ proper grip; see Figure 7.

Two inductive displacement sensors (IDS) were attached laterally to the specimens, through an aluminum frame. The frame was attached to the anchorage parts, and the IDS were installed to measure the elongation of the textiles. At the same time contrast markers were glued at both ends of the textiles for the purpose of assessing the elongation of the textile yarns by means of optical monitoring and subsequent DIC; see Figure 7b. Images with a resolution of 4024 × 6048 pixels were taken with a Nikon Z6 camera (Minato, Japan) at intervals of 2 s. The deformation histories of the yarns were evaluated using Aramis software by GOM GmbH (Braunschweig, Germany). The average deformation of three groups of markers was calculated to define the elongation of the entire textile. Note that in the GOM software the user defines the pairs of points to be tracked. The software determines automatically their relative spacing according to a local coordinate system (in pixel or in a length unit) and derives the resulting strain. This was done for every pair of markers on single yarns and averaged for every textile specimen. The slightly varying distances between the markers induced no error in strain calculation.

The measurements by the IDS in preliminary investigations yielded higher degrees of elongation than the ones resulting from the optical measurements, which could be attributed to the delamination and partial slip of the yarns from the anchorage, and to the micro-cracking occurring in the HS-SHCC pieces at the exit points of the textiles. For this reason, the elongation of the textile specimens as presented here were derived exclusively using the optical measurements and DIC. The IDS were used to measure the deformations of the composite specimens, as presented in the next section.

### 3.3. Testing Configuration for Composite Specimens

The specimens of plain HS-SHCC and of the hybrid fiber-reinforced composites were cast in the same mold as the textile specimens, without isolating the gauge portions. Taking into consideration the effective yarn cross-section given in Table 1, the longitudinal reinforcing ratio in the gauge portion of the specimens with textile reinforcement was 0.91%.

**Figure 8:**
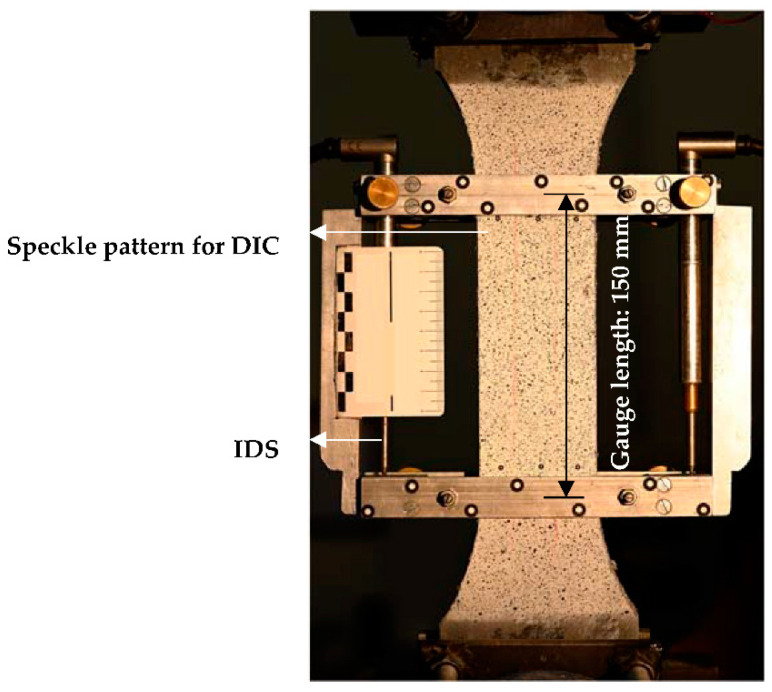
Tension testing configuration for composite specimens.

The uniaxial tension experiments were performed in the same testing configuration as for the textile specimens, at a displacement rate of 0.05 mm/s. In the case of the composite specimens, the elongation of the 150 mm-long gauge portion was measured by two IDS laterally on the specimens, as presented in Figure 8. A speckle pattern was sprayed on the observed surface of the specimens for optical monitoring and subsequent crack analysis by means of DIC.

### 3.4. Optical Measurements and Crack Analysis

Monocular image sequences were recorded during the tests, and subsequently analyzed using photogrammetric crack detection techniques [31,32,33]. The image processing chain begins with the definition of a grid of image points in the initial, i.e., unloaded state, and is followed by the computation of displacement fields to the following images using matching techniques with subpixel accuracy [36]. In the previous studies [26,35], the degree of multiple cracking and crack width were monitored and analyzed using Aramis software, by GOM GmbH. In that case the individual cracks needed to be defined by the user and measured manually, which was extremely tedious and time consuming, considering the large number of cracks and the epochs to be analyzed up to failure localization. To automatize this process, and provide a detailed statistical evaluation of the crack widths and numbers in all the pictures (epochs) taken, a dedicated DIC code was developed.

**Figure 9:**
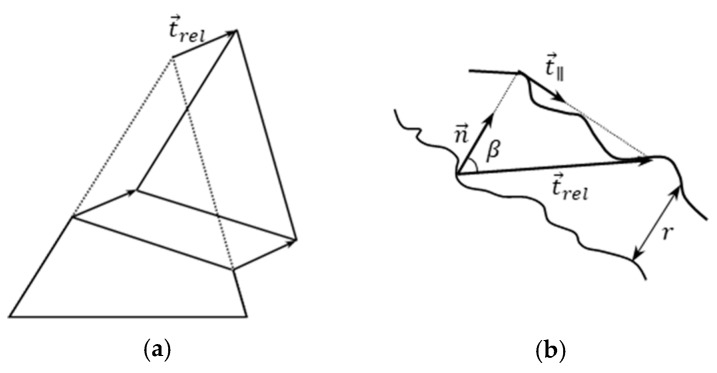
(**a**) Relative translation vector, and (**b**) components of ${\vec{t}}_{\mathrm{rel}}$, $\vec{n}$ is the crack normal, ${\vec{t}}_{\left|\right|}$ is the direction of the shear component; according to [26].

The evaluation principle was presented in detail in [31,32], and relies on the discontinuities generated by the cracks in the displacement fields. These are detected by triangle mesh geometry analysis after the triangulation of the matching points. The principal strains for each triangle are calculated to detect deformations, and define another deformation quantity: the length of the relative translation vector $\left|\left|{\vec{t}}_{\mathrm{rel}}\right|\right|$, where a triangle split and a parallel translation is assumed in the model as shown in Figure 9a. In case of noise in the field of $\left|\left|{\vec{t}}_{\mathrm{rel}}\right|\right|$, filter methods may be applied [33]. The technique allows for subpixel-accuracy in the detection of cracks, and the determination of their width. It is also possible to transform the length of the relative translation vector $\left|\left|{\vec{t}}_{\mathrm{rel}}\right|\right|$ into the object space using an image scale factor. ${\vec{t}}_{\mathrm{rel}}$ is composed of a component perpendicular to the crack (crack width *r*) and a shear component; see Figure 9b. The crack width is computed by the projection of ${\vec{t}}_{\mathrm{rel}}$ onto the crack normal, which is also estimated as shown in [31]. In certain cases the norm of the relative translation vector $\left|\left|{\vec{t}}_{\mathrm{rel}}\right|\right|$ is used as an approximation of crack width, especially if shear components are insignificant, which is generally the case for uniaxial tension experiments.

The multiple crack patterns were analyzed automatically based on three longitudinal profiles along the analyzed gauge portion of the loaded specimens. The discontinuities (cracks) analyzed crossing the profiles were captured, quantified, and in the case of cracks crossing all three profiles, averaged. This method is generally applicable in tension experiments on SHCC and TRC, due to the steady-state character of the cracks, i.e., instant propagation throughout the cross-section shortly after initiation. The code automatically provides the mean, the maximum, and the median of the crack widths, as well as the number of cracks for every epoch, and thereby enables detailed statistical analysis. In the current work, the mean crack widths and the number of cracks detected are presented in conjunction with the corresponding stress-strain relationships of the tested specimens.

## 4. Results and Discussion

### 4.1. Single-Yarn Pullout Tests

**Figure 10:**
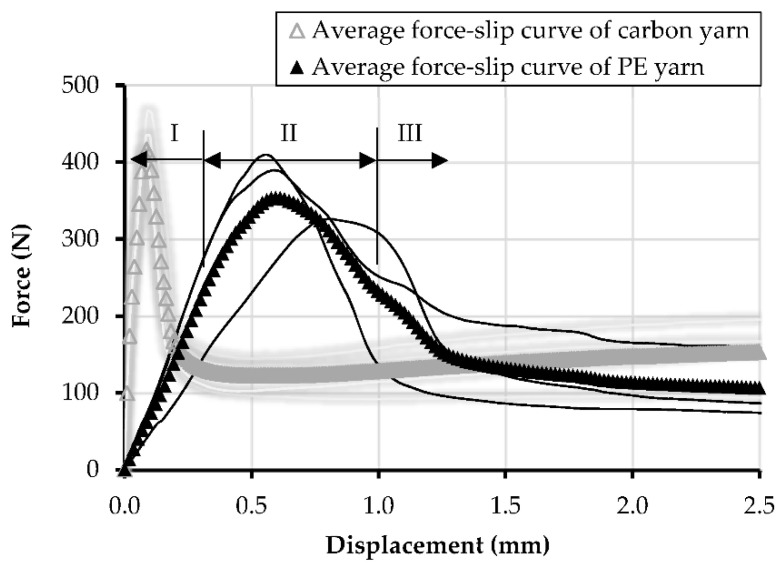
Force–displacement relationships and averaged curves for single PE yarns and equivalent carbon yarns, with an embedded length of 30 mm in HS-SHCC. The results of carbon yarns are adopted from [25].

**Figure 11:**
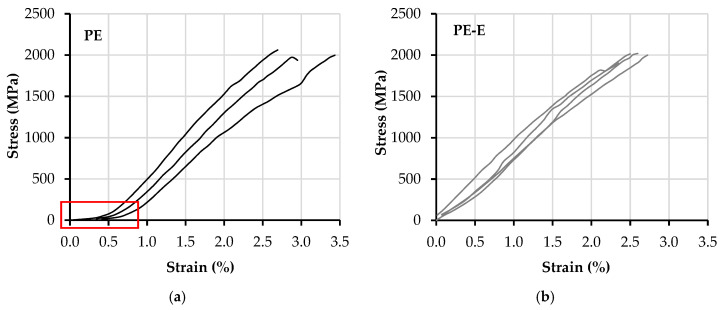
Tensile stress–strain curves obtained from the uniaxial tension tests on bare textile specimens (**a**) PE and (**b**) PE-E.

The force–displacement relationships of single PE yarns with an embedded length of 30 mm are presented in Figure 10. The displacement histories correspond to the average values of two LVDTs. The average peak force and the corresponding displacement are given in Table 3. The average bond strength was calculated by dividing the peak force by the contact surface area between yarn and matrix. The latter was derived based on digital microscopy of transverse slices extracted from the corresponding specimens. To compare, the curves of the single carbon yarns are presented in a shaded gray color, as adopted from the previous study by the authors [25]. Disregarding the type of yarn material, three typical pullout stages can be defined according to [37], namely: stage I—linear elastic, stage II—debonding, and stage III—dynamic pullout; see Figure 11. As seen in Figure 10, the chief, most obvious difference between the two types of yarns is related to the ascending slope of the controlled, pre-peak debonding phase, which is considerably shallower for PE textile when compared to carbon. The average peak pullout force was 375 N, which is also considerably lower than the average value corresponding to the carbon yarns in the previous study, which was of 440 N [25]. This might be partly related to the different impregnation quality. Additionally, considering the larger cross-sectional perimeter, due to the less compact arrangement of the single PE filaments, the difference between the derived bond strength is even higher, with 1.3 MPa for PE (Table 3) and 2.0 MPa for the carbon yarns; see [25].

Another aspect that influences the efficiency of shear force transfer from yarn to matrix is the relative displacement of the single filaments under load. With styrene-butadiene impregnation, the outer filaments of the yarns show a direct interaction with the surrounding matrix, while the inner filaments tend to slip against the outer sleeve filaments [38,39].

Preliminary pullout investigations on PE yarns with epoxy resin and sand coating were performed with varying embedded lengths, as low as 10 mm. These demonstrated repeatedly the failure of the HS-SHCC embedment piece, or of the yarns themselves, indicating substantially increased bond strength. However, the considerable increase in bond strength did not allow for a quantitative evaluation, this being the reason why no such experiments are presented here.

### 4.2. Uniaxial Tension Tests on Textile Specimens

The quasi-static tensile stress–strain relationships of the bare textiles, PE and PE-E, are presented in Figure 11. The stress histories were derived from the effective cross-sectional area of single yarns, as shown in Table 1, i.e., 2.18 mm^2^ × 5 yarns. The strain histories were derived through use of DIC, and using Aramis software, by GOM GmbH. Figure 11a indicates an obvious slacking effect of the PE textile. This is a result of the structural contraction of the multi-filament yarns during textile production; see Figure 1. This can be also judged by the low packing density of the single filaments inside the yarns, as shown in Figure 2b. The slacking effect receded considerably with the additional coating of epoxy resin and sand of PE-E. It seems that the latter ensures rigid confinement of the PE yarns pre-stretched during coating application, which explains the slightly lower strain capacity of PE-E compared to the reference PE textile.

The average tensile strength, strain capacity, and peak load of the textiles are summarized in Table 4. It can be seen that the additional coating of epoxy resin and sand did not affect the mechanical performance of the PE textile substantially. Compared to the carbon textile, with a strain capacity of 1.4% and tensile strength of 1638 MPa [26], PE textiles demonstrated higher tensile strength and almost double the strain capacity. It was, however, lower than the elongation of the single filaments at rupture, which is 3.5% according to the producers. Similarly, the tensile strength of approx. 2000 MPa was lower than that of the single filaments, as provided by the producers (>3000 MPa), which can be traced back to the non-uniform stretching of the single filaments inside the loaded yarns.

As mentioned in [26] and in Section 3.2, the results recorded by IDS included the deformation caused by yarn delamination and partial pullout from the cementitious anchorage pieces. The corresponding tensile stress–strain relationships of carbon textiles in [26] were not included in Figure 11, since in this work the deformations were recorded by an optical measuring principle.

### 4.3. Uniaxial Tension Tests on HS-SHCC

**Figure 12:**
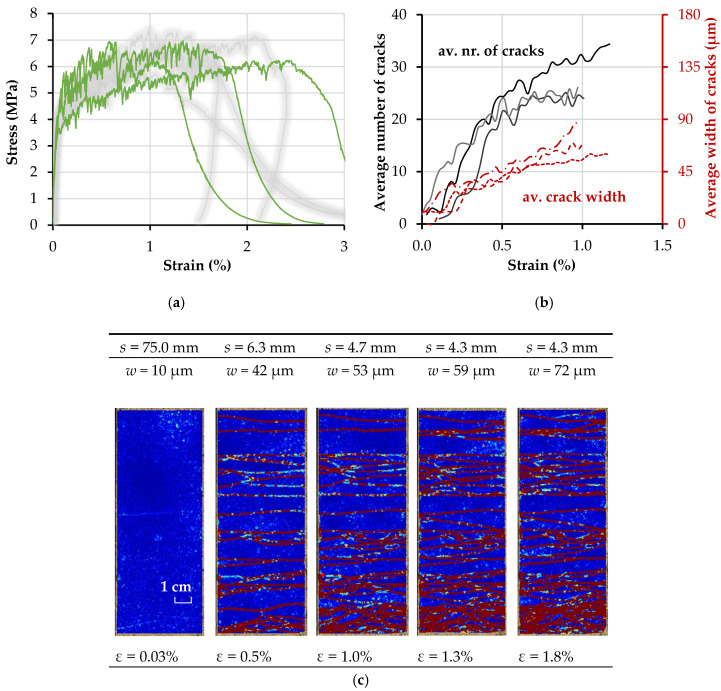
(**a**) Stress–strain curves, and (**b**) evolution of the average number of cracks and average crack widths with increasing deformation of the HS-SHCC specimens; (**c**) average crack spacing in representative HS-SHCC specimens, based on a sequence of DIC resolved images.

Figure 12a presents the stress–strain curves for the high-strength SHCC as green curves, together with the results obtained using specimens made of an identical HS-SHCC, as presented in the previous study (gray curves) [26]. Note that SHCC’s tensile behavior is strongly sensitive to any deviation in the mixture design and production process. The repeated production and investigation of SHCC specimens, additionally to the previous study, was motivated by the usage of new batches of constitutive materials (cement, silica fume). Besides the considerable scattering of the strains at failure localization, the results of both studies show on average no significant differences.

The tensile strength of the HS-SHCC and of the composites presented in the next section was derived as the ratio of peak load to the cross-sectional area of the corresponding specimens in the gauge portion. The latter deviated slightly from the nominal dimensions of 60 mm × 20 mm. The strain histories were derived as the average measurements of the IDS divided by the gauge length of 150 mm. The notion of strain in the case of SHCC indicates the global deformation as a result of the multiple cracking occurring in the monitored length, and it is only valid up to crack localization (initiation of softening). The softening branches of the stress–strain curves in Figure 12a are only presented for a description of the global material behavior and tensile failure.

The evolution of average crack widths and the number of cracks with specimen elongation in the gauge portion up to peak load is presented in Figure 12b. Note that both sets of curves show considerable unsteadiness, which is traced back to the relaxation occurring in the specimen with the formation of every new crack, causing partial closure of the existing cracks in deformation-controlled experiments. Whereas this can explain the negative trends of the crack width curves, the apparent reductions in the number of cracks are related to the fact of some cracks’ being close to levels below the threshold of crack detection, such that the DIC code automatically excludes them from the evaluation. Furthermore, if the spacing between the cracks becomes extremely small, e.g., below 2 mm, the algorithm cannot distinguish among individual cracks. This is, however, only an artifact of the DIC analysis. The accuracy of the presented DIC method allows the detection of cracks with a width of about a tenth of a pixel. The DIC method is an area-based algorithm, and cracks may largely influence the algorithm if they cross the surrounding areas of the grid points of the displacement field. Furthermore, an undeformed mesh triangle should lay between neighboring cracks. As a result, the spacing between neighboring cracks should be greater than double the size of the patch size added to the grid size to distinguish neighboring cracks. A patch size of 19 × 19 pixels, matching the resolution of 4024 × 6048 pixels of the camera, was used. The grid size was set to 5 × 5 pixels. The authors used a threshold for the deformation quantity of 32 pixels that corresponds to approximately 2 mm in the object space. The authors avoided smoothing the curves and excluding the negative trends, in order to present the actual output of the optical analysis.

The average tensile strength and strain capacity of the HS-SHCC was 6.7 MPa and 1.4%, respectively, as summarized in Table 5. Note that the strain capacity of the HS-SHCC was actually lower than anticipated, and it was also lower than the strain capacity of the PE textile. The relatively low strain capacity can be traced back to the relatively large specimen size, and to the semi-rigid boundary conditions. As shown in the previous work by the authors [26], the continuous reinforcement in such SHCC specimens confine them against in-plane rotation, in this way facilitating steady-state cracking, and considerably higher strain capacities compared to plain SHCC.

The average crack spacing “*s*” was calculated based on the number of cracks distributed along the 150 mm-long gauge length. The average crack width “*w*” of HS-SHCC prior to crack localization was 88 µm, with a standard deviation of 20.6 µm (Table 6), which is comparable to the measurements in previous work [21].

Note that plain HS-SHCC shows rather low robustness in terms of its strain capacity, which is a characteristic feature of these composites. Although the intrinsic material inhomogeneity, in terms of flaw content and flaw size, as well as fiber distribution, can be improved to a certain extent under laboratory conditions, in real-scale applications this issue can only be controlled to a limited extent. Besides providing only an uncertain basis for deriving nominal material properties for design purposes, this aspect indicates clearly the necessity for suitable, continuous reinforcement in strengthening layers made of SHCC to control the safe margin and reliability of the design material parameters.

### 4.4. Uniaxial Tension Tests on Hybrid Fiber-Reinforced Composites

**Figure 13:**
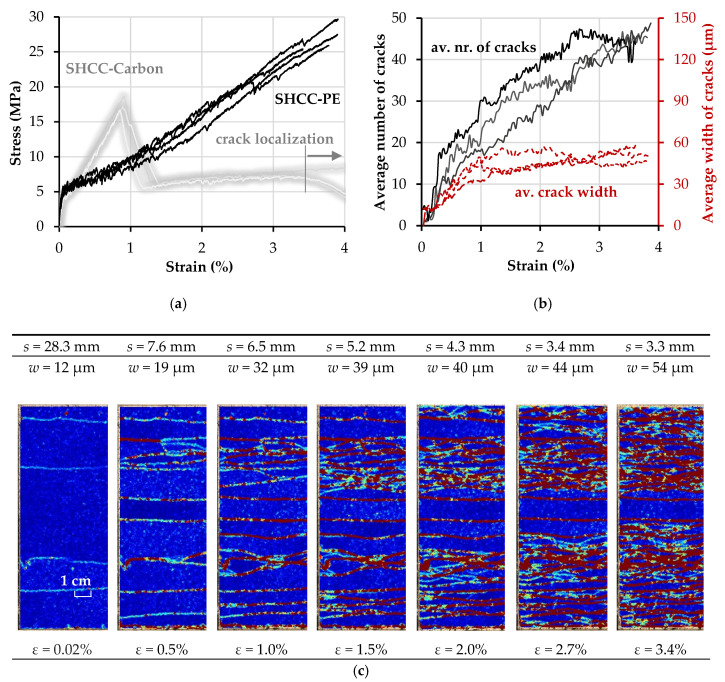
(**a**) Stress–strain curves, and (**b**) evolution of the average number of cracks and average crack widths with increasing deformation of SHCC-PE; (**c**) average crack spacing in a representative SHCC-PE specimen based on a sequence of digital image correlation (DIC) resolved images.

**Figure 14:**
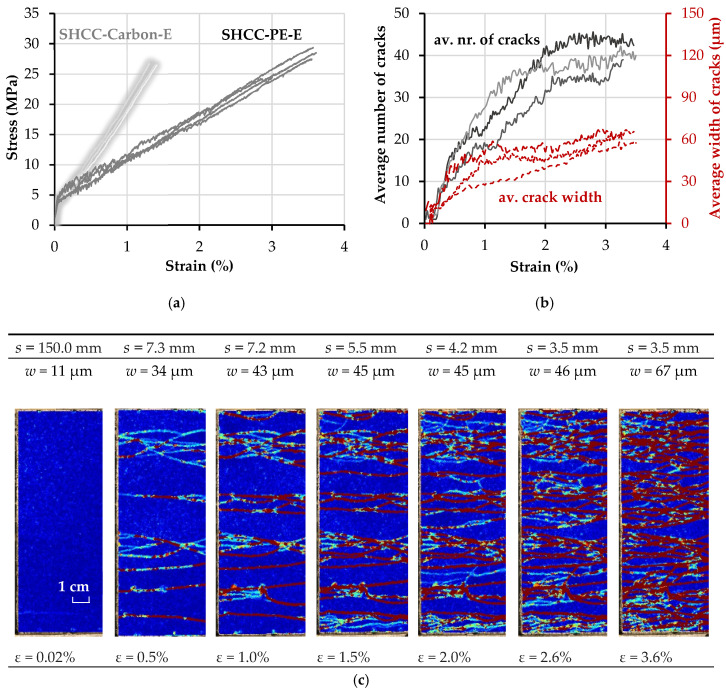
(**a**) Stress–strain curves, and (**b**) evolution of the average number of cracks and average crack widths under increasing deformation of SHCC-PE-E; (**c**) average spacing in a representative SHCC-PE-E specimen based on a sequence of DIC resolved images.

The tensile stress–strain curves, as well as the evolution of crack spacing and width in relation to the strain histories of the hybrid fiber-reinforced composites SHCC-PE and SHCC-PE-E are presented in Figure 13 and Figure 14. The tensile stress–strain curves of SHCC reinforced with carbon textile from the previous study [26] are plotted as shaded gray curves for comparison.

As opposed to plain HS-SHCC, the composites with textile reinforcement showed high robustness (low scattering) in terms of their elongation capacity, as dictated by the PE textiles. Moreover, both hybrid fiber-reinforced composites yielded a smaller average crack width prior to failure localization, compared to the plain HS-SHCC; see Table 6.

The composites reinforced with clean and extra-coated PE textiles exhibited comparable tensile strength and strain capacities; see Table 5. The stress–strain curves of SHCC-PE-E show a steady increase in tensile stress, along with increasing strain, as shown in Figure 14a, while the tensile behavior of SHCC-PE was influenced by the slacking of the PE textile, as can be judged by the shapes of the curves in the strain-hardening phase. The effective activation of the PE textile later in the strain-hardening phase does not allow for its full engagement in the load-carrying process in the initial load stage, and leads to a slight change in the slope of the ascending branch; see Figure 13a.

Unlike typical TRC, which exhibit only a limited number of cracks at low deformations, followed by steady crack growth with ongoing textile elongation [11,12,40], the multiple cracking spans of SHCC-PE and SHCC-PE-E continued up to strain values of at least 2.0% and higher. Actually, no obvious crack localization could be observed optically in the loaded specimens at higher deformations, which is also confirmed by the crack width curves in Figure 13b and Figure 14b. Note that the increased bond strength between the coated PE yarns and the surrounding HS-SHCC did not ensure more pronounced multiple cracking, or reduce the average crack width. On the contrary, at strains higher than 2%, the cracks of SHCC-PE-E yielded increasing crack openings, leading to a larger average crack width of 68.3 µm, compared to the 55.7 µm of SHCC-PE. The reason underlying this might be related to the different mechanisms of interaction between yarns and HS-SHCC, depending on their surface properties. In the case of the PE-E yarns, the bond between the epoxy resin and the PE yarns failed at larger crack openings, which could cause pronounced localized deformations in the yarns and, consequently, in the surrounding HS-SHCC.

The strain capacity of SHCC-PE was more than double that of HS-SHCC with carbon reinforcement, and the average crack width was slightly higher: 55.7 µm vs. 48.5 µm [26], which is attributed to the lower Young’s modulus and higher elongation on the PE yarns’ breaking. Although SHCC-PE yield higher work-to-fracture values, the energy dissipation capacity of SHCC-carbon is considerably higher at lower deformations, due to the higher stiffness of the carbon yarns. Note that, as shown in the shaded curves in Figure 13a, in the case of carbon textile with styrene-butadiene impregnation, the rupture of the carbon yarns in the HS-SHCC did not cause failure localization, and it continued to deform up to strain levels considerably higher than that of the plain HS-SHCC [21]. This is explained by the relatively weak bond and by the complete delamination of the carbon yarns from the surrounding HS-SHCC on yarn failure. Complete delamination was assumed based on the pronounced multiple cracking at that stage. Nevertheless, the ruptured carbon yarns in combination with the low frictional bond confined the specimens structurally, with ongoing global elongation of HS-SHCC and pullout of the carbon yarns, as well as impeded premature crack localization. On the contrary, the carbon yarns, with an additional coating of epoxy resin and sand, ensured a strong composite action and crack localization immediately after yarn failure; see gray curves in Figure 14a.

Compared to the equivalent composites reinforced with carbon textile, SHCC-PE exhibited a pronouncedly denser crack pattern, with an average spacing of 3.3 mm compared to 5.2 mm in the previous study [26]. The higher strain capacity of PE textile ensures superior deformational compatibility with the HS-SHCC, and leads to considerably higher work-to-fracture values up to failure localization. Whether this large deformability can be exploited in real applications depends on the loading scenario, e.g., impact and blast, and on the resilience and safety requirements of the impacted structural components [1].

## 5. Conclusions and Outlook

As a continuation of the previous investigation by the authors on carbon textile [26], the article at hand introduced a new UHMWPE textile, which was produced specifically for this study, and which exhibited comparable tensile strength, but considerably higher elongation capacity. The presented study emphasizes the influence of the textile material and surface properties on the composite tensile behavior in combination with HS-SHCC. Quasi-static single-yarn pullout tests and uniaxial tension tests on UHMWPE textile, plain HS-SHCC, and hybrid fiber-reinforced composites are presented and discussed. An optical measuring principle was adopted for deriving the elongation of the textiles, which allowed excluding the deformation caused by yarn delamination and pullout from the cementitious anchorage pieces. A dedicated DIC code was developed using photogrammetric crack detection techniques for automatically analyzing the crack widths and spacing.

In comparison to plain HS-SHCC, continuous textile reinforcement facilitates not only a considerable increase in tensile strength, but also superior strain capacity and crack control, as well as enhanced robustness. The use of PE textile contributed to higher work-to-fracture of the composites compared to those made with carbon textile [26], thanks to the higher elongation capacity of the former. Furthermore, instead of yarn failure before reaching crack saturation in SHCC, the high elongation capacity of the polymer textile enabled a more pronounced development of multiple cracks prior to yarn failure. However, it should be noted that, if assessed at lower strain values, SHCC reinforced by carbon textile prevails in terms of load-carrying capacity and work-to-fracture, due to the higher Young’s modulus of the carbon yarns [26]. Hence, the combination of HS-SHCC with UHMWPE textile reinforcement might be more suitable when extremely large inelastic deformations of the composite are required.

The application of epoxy resin and sand coating on the polymer textile did not improve the multiple cracking behavior of the hybrid fiber-reinforced composites. However, the additional coating contributed to a more rigid textile structure, reducing the slacking effect in the initial loading phase, and an earlier activation of the textile in load bearing process in hybrid fiber-reinforced composites. Additionally, proper textile stretching before production mitigated the slacking effect.

Note that the presented studies have only focused on the tensile properties of the composites. In ongoing studies, the effects of different textile types on the synergetic action of hybrid reinforced composites are being investigated under shear and dynamic loading. Furthermore, numerical parameter studies involving stochastic material properties are being performed within the framework of finite element modelling, targeting data-driven material design and a more detailed assessment of various micromechanical and structural parameters, and their effect on the tensile behavior of hybrid, fiber-reinforced composites, both at the meso-scale, and as strengthening layers in structural elements.

## Figures and Tables

**Table 1:** Geometric properties of the ultra-high molecular weight polyethylene (PE) textile.

Parameter	Warp Yarn	Weft Yarn
Average yarn count (tex)	2112	528
Effective yarn cross-section (mm^2^)	2.180	0.545

**Table 2:** Mixture composition of the high-strength strain-hardening cement-based composite (SHCC) under investigation.

Components	Content in (kg/m^3^)
CEM I 52.5R-SR3/NA (Holcim, Rapperswil-Jona, Switzerland)	1460
Elkem 971-U silica fume (Elkem, Oslo, Norway)	292
Quartz sand 0.06–0.2 mm (Strobel Quarzsand GmbH, Freihung, Germany)	145
Superplasticizer Glenium ACE 460 (BASF, Ludwigshafen am Rhein, Germany)	45
Water	315
UHMWPE SK62 fiber—2% vol. (DSM, Heerlen, the Netherlands)	20

**Table 3:** Average values of the pullout parameters, with standard deviations given in parentheses.

Textile Material	Peak Pullout Load (N)	Slip at Peak Load (mm)	Average Bond Strength (MPa)
PE	375.0 (43.5)	0.8 (0.1)	1.3 (0.2)

**Table 4:** Average tensile properties of the textiles under investigation, with standard deviations given in parentheses.

Parameter	PE	PE-E
Strain capacity (%)	3.0 (0.4)	2.5 (0.2)
Tensile strength (MPa)	2011.3 (45.5)	1984.3 (55.3)
Peak load (kN)	21.9 (0.5)	21.6 (0.6)

**Table 5:** Average tensile properties of the composites under investigation, with standard deviations given in parentheses.

Composite	First-Crack Stress (MPa)	Tensile Strength (MPa)	Strain Capacity (%)	Work-to-Fracture (kJ/m^3^)	Peak Load (kN)
SHCC	2.7 (1.3)	6.7 (0.4)	1.4 (0.9)	79.2 (47.4)	7.9 (0.4)
SHCC-PE	3.3 (1.1)	27.1 (1.9)	3.8 (0.2)	570.6 (70.2)	29.2 (1.3)
SHCC-PE-E	2.5 (0.8)	27.5 (2.1)	3.4 (0.3)	547.2 (64.2)	30.0 (2.2)

**Table 6:** Average crack spacing and crack width in the investigated composite specimens prior to failure localization, with standard deviations given in parentheses.

Parameter	SHCC	SHCC-PE	SHCC-PE-E
Average crack spacing *s* (mm)	5.3 (0.9)	3.0 (0.2)	3.5 (0.2)
Average crack width *w* (µm)	88.3 (20.6)	55.7 (3.3)	68.3 (2.0)
